# Electrocoagulation for Polystyrene and Polyethylene Microplastic Removal: Particle Size Effects, Mechanisms, and Floc Characterization

**DOI:** 10.1021/acsestwater.6c00398

**Published:** 2026-07-28

**Authors:** Sara Mateo, Jannis Wenk, John Chew, Antonio Exposito

**Affiliations:** † Department of Chemical and Materials Engineering, Complutense University of Madrid, Madrid 28040, Spain; ‡ Department of Chemical Engineering, 1555 University of Bath, Bath BA2 7AY, U.K.; § Division G - Qualitative Hydrology, Department G1 - General Water Quality Issues, Federal Institute of Hydrology, Koblenz 56068, Germany

**Keywords:** microplastics, polystyrene, polyethylene, electrocoagulation, wastewater, flocs

## Abstract

Microplastics (MPs) have emerged as a critical environmental issue, with electrocoagulation showing promise as an efficient technology for MP removal in wastewater treatment. However, there is a limited understanding of how the electrocoagulation process parameters influence removal efficiency across different MP types and sizes. This study addresses this gap by investigating the effects of the MP type and particle size (43–125 μm) on MP-floc aggregate evolution and their interactions, which affect the mechanism and efficiency of removal, using a batch reactor with a sacrificial iron anode under galvanostatic conditions (16.7 A m^–2^). MP removal efficiencies reached up to 81% for polystyrene (85–105 μm) and 79% for polyethylene (106–125 μm). Samples were collected at discrete intervals to monitor the process through multi-image capture and analysis. The work elucidates how the MP surface hydrophilicity and molecular structure govern interactions with coagulants, affecting dominant removal mechanisms (polystyrene: sweep flocculation; polyethylene: charge neutralization) and energy requirements. Zeta potential and FTIR analyses revealed surface modifications and electroactive species formation. Floc characterization showed that polystyrene forms compact, elongated flocs, whereas polyethylene produces more spongy, symmetrical aggregates. Floc size correlated positively with MP size. These mechanistic insights advance the understanding of MP–coagulant interactions and provide a framework for optimizing reactor design.

## Graphical abstract

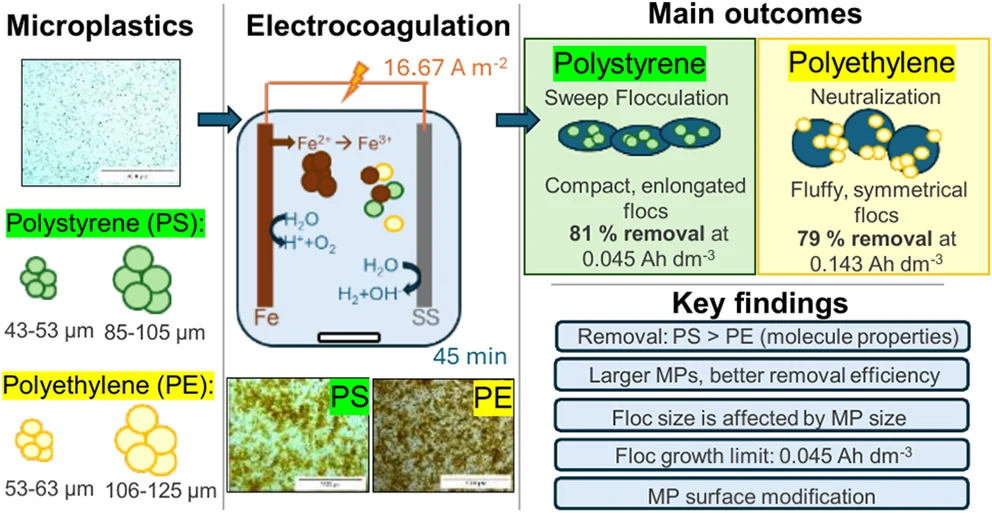

## 1 Introduction

Global plastic production and consumption have rapidly increased, dominated by nonbiodegradable, single-use plastics.^1^ To address the environmental risks of plastic pollution, tracking plastic debris has become a key metric in international marine protection efforts,^2^ alongside policy initiatives worldwide aimed at reducing plastic and microplastic pollution. Examples include the Global Plastics Treaty, the UK’s 25-year Environment Plan, the EU’s 2030 zero-waste strategy and Single-Use Plastics Directive, and regional efforts such as the Pacific Alliance’s sustainable plastic management.^3–5^ Despite these efforts, plastic production continues to grow exponentially, rising from 299 million metric tons (MMT) in 2011 to over 413 MMT in 2023.^6^ Only about 9% of the plastics were effectively recycled between 2019 and 2022 (1), while approximately 2.5% enter the oceans,^7^ eventually breaking down into persistent microplastics (MP, 1 μm to 5 mm)^8^ and nanoplastics (1 nm to 1 μm).^9^

Major sources of MPs include cigarette filters, synthetic textiles, cosmetics, food packaging, fishing debris, and plastic bags.^10,11^ The most prevalent polymers in the environment are polystyrene (PS), polyethylene (PE), and polypropylene (PP).^12^ These plastics resist biodegradation,^13^ have densities close to water,^14^ and undergo surface modifications from environmental stressors such as UV radiation, abrasion, and chemical oxidation.^15^ Moreover, additives such as bisphenol, phthalates, and polybrominated diphenyl ethers can leach from plastics during degradation.^13,15,16^ MPs have been detected in aquatic ecosystems,^10^ ingested by marine life,^12^ and even found in human blood and placenta, posing risks of pulmonary, intestinal, immunotoxic, and cardiovascular diseases and cancer.^17–20^

Given the environmental and health implications, research on MPs has intensified across three main fronts: standardized quantification methods, studies of MP environmental behavior, and development of effective removal technologies. Current removal strategies span conventional treatments—such as the activated sludge processes, coagulation, and rapid sand filtration—to advanced methods including membrane bioreactors, dissolved air flotation, and magnetic separation. Emerging approaches including adsorption via hybrid silica gels, photocatalytic TiO_2_ micromotors, and algae biofiltration are also being explored, alongside degradative pathways like photocatalysis and electrooxidation.^16,20–22^

Electrocoagulation (EC) has attracted interest for MP removal due to its high efficiency, operational simplicity, and environmental benefits. EC operates by electrodissolution of a sacrificial anode—commonly iron or aluminum—under applied current, producing coagulant species that aggregate contaminants into flocs for sedimentation. These coagulants, primarily insoluble metal hydroxides and hydroxy complexes of the anode material, function similarly to those in conventional coagulation (CC) by adsorbing contaminants or neutralizing particle surface charges to promote aggregation. Compared to CC, EC reduces chemical usage, produces less and more manageable sludge, enhances flocculation through oxygen and hydrogen bubble generation, shortens treatment times, and offers improved feasibility, cost-effectiveness, and sustainability.^23–27^

Most studies on EC for MP removal focus on cell design and optimization of operational parameters. Table 1 summarizes these studies outlining parameters driving removal efficiency—anode material and initial pH, which determine coagulant chemistry and metal speciation; driving force, which governs coagulant dosage; and MP characteristics, which affect available surface area for adsorption. Outcomes are detailed in terms of maximum removal efficiency and corresponding treatment time.^28–38^ Operational parameters and cell design define floc properties and MP–floc interactions, both of which determine the overall removal efficiency.

**Table 1:** Summary of Electrocoagulation Studies for Microplastic Removal, Detailing Reactor Configurations, Wastewater Types, Microplastic Characteristics, Operational Parameters, Removal Efficiencies, and Treatment Durations

process	A-C^a^; gap (cm)	WW^a^	MP; size (μm); [MP]	driving force	pH	RP (%)	Rt (min)	ref
EC/EF	Al–Fe; 0.2	SWW^a^	PE; 150; 0.2 MPs/mL	15 A/m^2^	7.0	100	20	^28^
EC/EF	Fe–Al; 0.2	SWW	PE; 150; 0.2 MPs/mL	15 A/m^2^	7.0	96	120	^28^
EC/EF	Fe–Al; 0.2	SWW	PVC; 250; 0.2 MPs/mL	20 A/m^2^	7.0	96	10	^28^
EC	Fe-SS; 3	SWW	PET; 150–250; 50 MP/L	15 V, 1 A	8.4	91	3	^29^
EC	Fe-SS; 3	SWW	LDPE; 150–250; 50 MP/L	15 V, 1 A	8.4	89	3	^29^
EC	Fe-SS; 3	SWW	PP; 150–250; 50 MP/L	15 V, 1 A	8.4	86	3	^29^
EC	Fe-SS; 3	SWW	PA; 150–250; 50 MP/L	15 V, 1 A	8.4	86	3	^29^
EC	Al–Cu; 2	SWW	PE; 286.7; 0.5 g/L	10 V	7.2	93	240	^30^
EC	Al–Cu; 2	SWW	PMMA; 6.3; 0.5 g/L	10 V	7.2	92	240	^30^
EC	Al–Cu; 2	SWW	CA; 1–2 mm; 0.5 g/L	10 V	7.2	98	240	^30^
EC	Al–Cu; 2	SWW	PP; 1–2 mm; 0.5 g/L	10 V	7.2	98	240	^30^
EC	Fe–Cu; 2	SWW	PE; 286.7; 0.5 g/L	10 V	7.2	72	240	^30^
EC	Fe–Cu; 2	SWW	PMMA; 6.3; 0.5 g/L	10 V	7.2	59	240	^30^
EC	Fe–Cu; 2	SWW	CA; 1–2 mm; 0.5 g/L	10 V	7.2	85	240	^30^
EC	Fe–Cu; 2	SWW	PP; 1–2 mm; 0.5 g/L	10 V	7.2	83	240	^30^
EC	Al–Al	SWW	PS 25–65 μm, 0.025 g/L	8.07 A/m^2^	7.0	99	60	^31^
EC	Bipolar Al–Al	RWW+MP^a^	PE; 100–200 μm	12 mA/cm^2^	6.6	98	30	^32^
EC	Fe–Al; 2.5	SWW	PA; 38–74 μm; 0.5 g/L	10 V	7.0	97	120	^33^
EC/EF	Fe–Al	RWW	15804 MP/L	2.16 A	9.0	98	60	^34^
EC	Al–Al; 1	SWW	PS;150–300 μm; 0.2 g/L	0.2 A	7.0	95	50	^35^
EC	Al–Al; 0.5	RWW	PE; 200–5000 μm; 86 MPs/L	3.81 mA/cm^2^	6.0	99	15	^36^
EC	Al-graphene	SWW	PET; 45–250 μm; 0.5 g/L	10 V	5.5	96	80	^37^
EC	Al-graphene	SWW	PP; 45–250 μm; 0.5 g/L	10 V	5.0	80	30	^37^
EC	Al-graphene	SWW	PS; 45–250 μm; 0.5 g/L	10 V	3.0	94	90	^37^
Pulsed EC	Ti–Al; 1	SWW	PS; 150 μm; 0.5 g/L	4 A/m^2^	7.0	93	60	^38^

a A-C: anode–cathode; WW: wastewater; j: current density; CP: cell potential; RP: removal performance; Rt: removal time; SWW: synthetic wastewater; RWW: real wastewater; RWW+MP: real wastewater artificially contaminated with MP; and EC/EF: electrocoagulation/electroflotation.

The growth factor dictates the floc’s evolutionary trajectory, where the resulting size and fractal dimension collectively determine the aspect ratio, resistance to shear, and settling efficiency by governing spatial arrangement and hydrodynamic drag. Floc size characterizes the aggregation stage, fractal dimension reflects geometric complexity and compactness, and the aspect ratio quantifies deviation from sphericity, directly impacting aerodynamic behavior and settling trajectory. These floc properties are influenced by coagulant mechanisms^39^ and, despite their combined importance for floc performance, are usually studied individually rather than simultaneously.^40^ While relationships among these factors (e.g., floc size and fractal dimensions) have been extensively studied in CC, similar research in EC remains limited,^40–42^ making comprehensive evaluation essential to clarify MP removal mechanisms.

Theoretical models propose that MP–floc interactions depend on factors such as MP size and floc size, with larger flocs incorporating MPs up to a size limit and undergoing fragmentation under shear.^43,44^ Most research has focused on activated sludge and CC flocs with model particles such as kaolinite,^45^ while EC floc characterization and MP interactions, despite being critical for advancing current wastewater treatment processes,^46^ remain underexplored.

This study aims to fill this gap by experimentally investigating the EC of MPs of different types (PS and PE) and sizes, focusing on EC parameter dynamics and mechanisms of MP-floc formation, development, and interactions. Using a batch EC reactor with a sacrificial iron anode, MP removal efficiency was measured alongside floc morphological parameters through multi-image capture and analysis. By characterizing flocs during both the growth and sedimentation phases, this research elucidates the morphological dynamics and sequestration kinetics of the process. The implications are 3-fold: (1) new insights into the influence of MP nature and size on EC performance and floc structural evolution during treatment; (2) a mechanistic framework for optimizing the reactor design based on floc kinetics and energy efficiency of MP removal, as well as for designing post-treatment stages based on the characterization and stability of the formed flocs; and (3) groundwork for predictive studies of MP behavior during full-scale water treatment.

## 2 Materials and Methods

### 2.1 Microplastic Suspensions

All chemical and analytical reagents were purchased from commercial suppliers. Experiments used aqueous suspensions prepared with ultrapure water containing 500 mg L^–1^ of various microplastic (MP) types, with 0.01 M NaCl as an electrolyte (Fisher Chemical, purity ≥ 99.5%) and 2.44 mg L^–1^ of the nonionic surfactant Polysorbate 20 (Sigma-Aldrich). The MPs selected were polyethylene (PE) and polystyrene (PS), chosen for their similar densities to water and availability. MPs were purchased from Cospheric: spherical PE particles sized 53–63 μm and 106–125 μm (density: 1.00 g/cm^3^) and spherical PS particles sized 43–53 μm and 85–105 μm PS (density: 1.07 g/cm^3^). Manufacturer particle size distributions were verified using a Malvern MasterSizer 3000 (Section 2.4).

The composition was selected based on a previous study that simulated MP hotspots.^47^ Although MP concentration is higher than typical environmental concentrations, this concentration exceeds the detection limits of some of the analytical techniques used and thus ensures an accurate mechanical characterization. The concentration of the electrolyte was established to ensure that the suspension had a conductivity similar to that of domestic wastewater. In addition, the controlled environment was essential for isolating and unambiguously identifying the specific behaviors of MP-floc aggregates and their interactions.

### 2.2 Electrocoagulation Setup and Operation

Electrocoagulation (EC) tests, each lasting 45 min, were conducted at the initial concentrations of MPs, electrolyte, and surfactant described above. The test batch reactor consisted of a 0.6 L beaker containing the suspension and submerged electrodes: a sacrificial iron (Fe) anode and a stainless-steel AISI 316L cathode (dimensions: 120 × 40 × 1 mm). The beaker was placed on a stirring plate set to 100 rpm. The EC process was operated at room temperature (18 °C) under galvanostatic conditions with a current density of 16.67 A m^–2^ and an interelectrode spacing of 40 mm.^47^ Repeatability was confirmed by performing experiments in triplicate. Blank EC experiments without MPs were also performed. An extra experiment under the same operation conditions and using PS of 85–125 μm was carried out to provide a reproducibility check. This independent verification was performed in triplicate to validate the findings.

### 2.3 Sample Collection

Samples were collected from the bulk reactor at discrete time intervals of 5, 10, 15, 20, 30, and 45 min. To ensure comparability with results from the literature under different operating conditions, these time points were directly correlated with specific electrical charge values of 0.011, 0.025, 0.045, 0.074, 0.143, and 0.300 Ah dm^–3^, respectively. The samples were analyzed to monitor the EC process by measuring the pH, conductivity, ζ-potential, and total iron concentration over time. After collection, the samples were allowed to settle for 1 h to separate phases, enabling independent analysis of the supernatant and settled solids.

### 2.4 Analytical Methods

pH and conductivity were measured using a VWR pHenomenal 1100L pHmeter and a VWR Avantor CO 3100 H conductivity meter. Dissolved iron (Fe) concentration from the anode was determined after diluting samples at 1:10 with ultrapure water and acidifying with HNO_3_ to pH < 2 (Thermo Scientific, purity >70%) to ensure iron solubilization, followed by filtration through a 45 μm filter to remove MPs. Fe concentrations were quantified by ICP-OES (Agilent 710 series) with an autosampler.

For MP removal analysis, the supernatant collected after 1 h of sedimentation was evaporated at 90 °C for 18 h to remove water. The dried residue was then dissolved in tetrahydrofuran (THF, Thermo Scientific, purity >99.8%) under gentle heating and stirring to ensure complete dissolution of MPs. MP concentrations were measured by UV–vis spectrophotometry (Agilent Cary 100) at 212 nm for PS and 226 nm for PE.^47,48^ MP removal (%) was calculated using

1 $$\mathrm{MP}\;\mathrm{removal}\;\left(\%\right)=\frac{C_{0,\mathrm{MP}}-C_{\mathrm{MP}}}{C_{0,\mathrm{MP}}}\times 100$$

where *C*_0,MP_ is the initial MP concentration in the bulk suspension and *C*_MP_ is the MP concentration in the supernatant after 1 h of sedimentation.

To determine the solid volume fraction involved in sedimentation, a known volume of the settled phase after 1 h was transferred in a preconditioned (90 °C, 2h) and preweighted empty vial and dried (90 °C, 18 h). The remaining solid was weighed to obtain the combined mass of Fe and MPs. Subtracting the MP mass in the supernatant yields the MP mass in the sludge; by difference, the Fe mass is obtained. The volume and volumetric fraction of both MP and Fe were calculated individually using their respective densities. For Fe species, density ρ_Fe(OH)_3__ = 3.4 g cm^–3^ was used, corresponding to Fe­(OH)_3_, the main Fe species produced during EC.

### 2.5 Particle Characterization

Zeta potential measurements were performed using a Malvern Zetasizer Ultra Analyzer (Model ZSU3305). Initial and final MP sizes, the latter measured from the supernatant after EC, were determined with a Malvern Mastersizer 3000 equipped with a Hydro SM manual liquid sample unit (Model MAZ3150) and an external control module for stirring and pumping (350–3500 rpm). Fourier transform infrared (FTIR) spectroscopy was used to detect chemical changes post-EC. Spectra of individual components (Fe, PS, PE) and final EC sludge samples (Fe-PS and Fe-PE) were recorded using a Bruker FT-IR spectrometer INVENIO. The Fe spectrum was obtained from sludge collected after evaporating water from the blank experiments (90 °C, 18 h), while PS and PE spectra were recorded from the native commercial MPs.

### 2.6 Image Acquisition and Analysis

Image acquisition and analysis were performed on agitated samples from the bulk and their supernatants after 1 h of sedimentation. Images were captured using a Thorlabs DCU224C camera equipped with an adjustable Navitar 12 × zoom lens, as described previously.^46,49,50^ The camera was mounted on a support system to ensure precise and consistent image capture. Samples were placed in a transparent methacrylate viewing cell (134 × 115 × 10 mm), positioned between a Nightsearcher Galaxy Pro light source (20 cm away) and the camera (5–10 cm away). Images were recorded at five frames per second, capturing up to 50 images per sample to minimize random variation. Before each image series, a scale image was taken to calibrate pixel-to-size conversion, with the scale (cm/pixel) calibrated prior to all image analyses. ImageJ software was used to measure the floc diameter, aspect ratio (longest side divided by the shortest side), and 2D fractal dimension using software’s Fractal Box Count tool.

### 2.7 Sedimentation and Particle Balance Calculations

The settling velocity of the particle suspension was calculated using the Richardson and Zaki equation^51^

2 $$u_{c}=u_{t}{\left(1-ø\right)}^{n-1}$$

where *u*_c_ is the settling velocity of the particle suspension (m/s), *u*_t_ is the terminal velocity of a single particle, *ø* is the solid volume fraction, and *n* is an empirical sedimentation exponent. Here, *n* was taken as 4.7, consistent with literature values for laminar to inertial flow regimes.^51,52^

The terminal velocity *u*_t_ was calculated using Stoke’s law^51^

3 $$u_{t}=\frac{g\cdot {d_{\mathrm{floc}}}^{2}\cdot {(\rho }_{\mathrm{floc}}-\rho_{\mathrm{water}})}{\mu }$$

where *g* is the gravity, *d*_floc_ is the floc diameter obtained by ImageJ (section 2.3), μ is the water viscosity, ρ is the density, and ρ_floc_ is the floc density calculated as:

4 $$\rho_{\mathrm{floc}}=\rho_{{\mathrm{Fe}\left(\mathrm{OH}\right)}_{3}}\cdot {ø}_{{\mathrm{Fe}\left(\mathrm{OH}\right)}_{3}}+\rho_{\mathrm{MP}}\cdot {ø}_{\mathrm{MP}}+\left(1-{ø}_{{\mathrm{Fe}\left(\mathrm{OH}\right)}_{3}}-{ø}_{\mathrm{MP}}\right)\cdot \rho_{\mathrm{water}}$$

Applicability of Stoke’s law was verified by calculating the Reynolds number (*Re*) (eq 5). In this work, *Re* < 0.1, and Stoke’s law applies without requiring velocity corrections.

5 Re=ρwater·ut·dflocμ

The floc growth rate was calculated as the difference in the floc diameter between two samples divided by the elapsed time.

## 3 Results and Discussion

### 3.1 Microplastic Removal

Figure 1 shows the evolution of MP removal (%) as a function of the applied electric charge (Q, Ah dm^–3^). For both MP types and size ranges, removal efficiency increased with increasing charge, reaching a maximum before slightly declining. Removal peaked between 0.045 and 0.143 Ah dm^–3^ and then leveled off at around 0.3 Ah dm^–3^. Maximum removal rates for PS particles were 73.0 ± 0.3% (43–53 μm) and 80.6 ± 4.5% (85–105 μm) at 0.045 Ah dm^–3^, corresponding to 15 min EC. For PE particles, the highest removal efficiencies occurred at higher charges: 71.8 ± 4.7% (53–63 μm) and 79.1 ± 3.3% (106–125 μm) at 0.143 Ah dm^–3^, equivalent to 30 min of EC. Overall, PS showed slightly higher removal than that of PE, and larger particles were removed more efficiently regardless of the polymer type. These trends align with previous findings and are attributed to reaching the effective EC window limit at high applied electric charges, beyond which efficiency decreases.^47^ Overall, the method demonstrates consistent performance across different MP materials and sizes, with small differences observed. The higher removal of PS may relate to greater surface hydrophilicity, which favors coagulation and removal, supported by contact angle measurements correlating hydrophilicity with removal yields.^53^ Particle size also influences removal, as larger particles enhance aggregation during EC and settle more readily. Similar MP removal rates have been reported using coagulation–flocculation with aluminum salts as the coagulant and polyacrylamide as the flocculant.^54^ For better understanding, images taken of the supernatant of the samples from the EC are shown in the SI (Figures 6S-9S).

**Figure 1:**
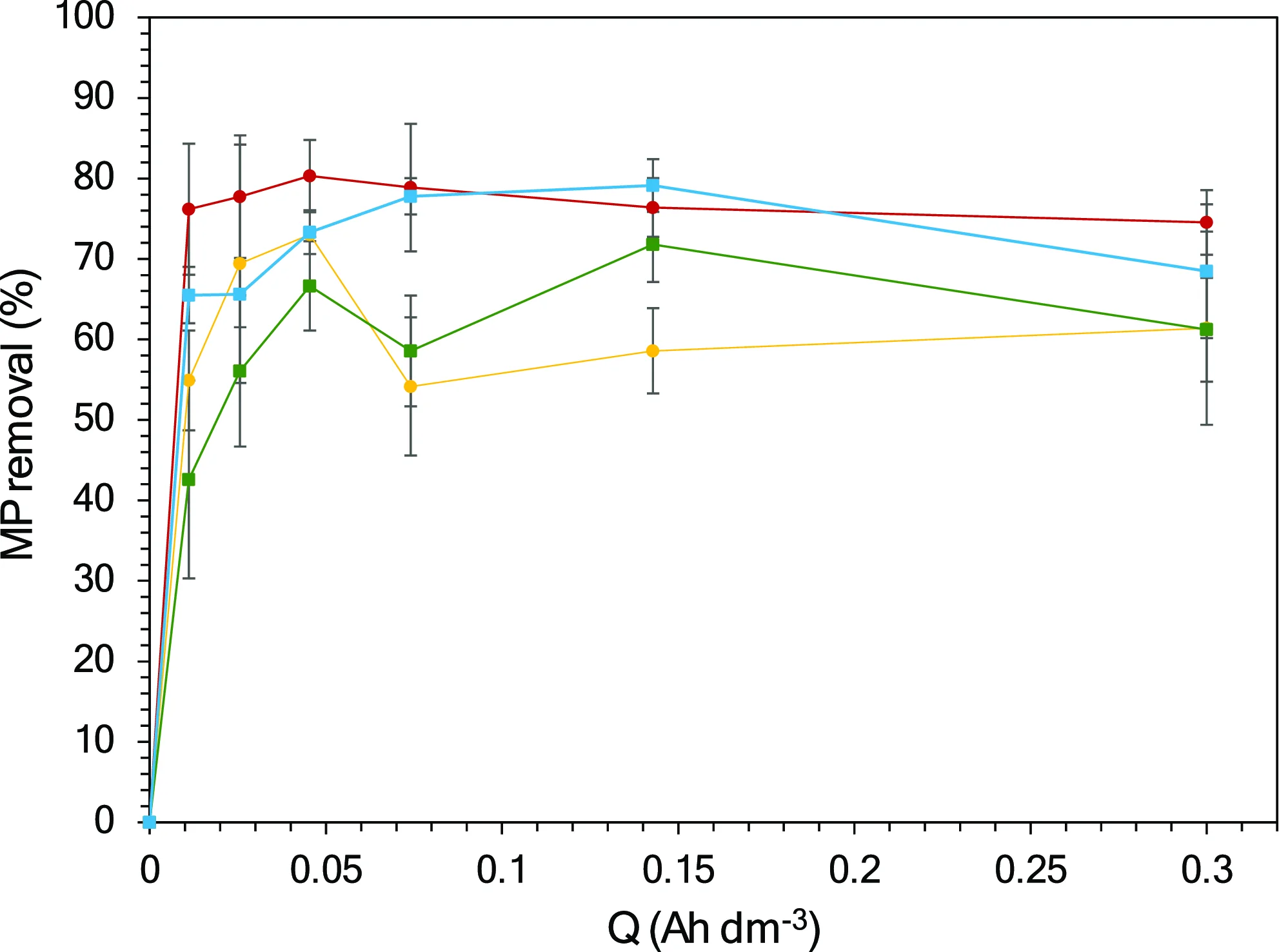
Evolution of MP removal (%) with the applied electric charge. (● yellow) PS 43–53 μm; (■ green) PE 53–63 μm; (● red) PS 85–105 μm; and (■ blue) PE 106–125 μm. Operating conditions: j: 16.67 A m^–2^; *C*_0 electrolyte_: 0.01 M; *C*_0,MP_: 0.5 g L^–1^; interelectrode gap: 40 mm; *V*_tank_: 0.65 L; treatment time: 45 min; and anode: Fe. Error bars represent the standard deviation of triplicate measurements.

### 3.2 Iron Dissolution Kinetics

Figure 2 shows the evolution of dissolved iron concentration released from the sacrificial anode^55^ as a function of applied electric charge. Iron release is rapid up to approximately 0.045 Ah dm^–3^, after which the dissolution rate slows, suggesting electrode passivation. This is supported by the sharp increase in cell voltage from 4 to 14 V observed in all cases, as well as trends in MP removal (Figure 1) and pH (SI Figure 1S).^56,57^ According to Faraday’s law,^58^ iron dissolution should be largely independent of the MP type and size.^59^ However, compared to blanks, iron dissolution kinetics are faster in PS-EC systems and slower in PE-EC systems. This difference may relate to the MP type: PS’s greater affinity for iron species, possibly due to its surface hydrophilicity and aromatic structure, can promote PS–coagulant interactions that favor the electrodissolution of the anode. In contrast, PE lacks aromatic rings, which may slow PE–coagulant interactions^60^ and contribute to anode passivation, as reflected by higher pH values for PE-EC systems (SI, Figure S1), ultimately affecting the primary MP–floc interaction mechanism.^57^ Comparison with MP removal data (Figure 1) suggests that maximum efficiency is achieved around 30–40 ppm dissolved iron, beyond which additional iron does not improve removal, indicating system limitations. The iron dosage aligns with literature values, ranging from approximately 20 ppm for wastewater treatment in CC^61,62^ to higher concentrations in EC applications, e.g., 30 ppm to remove Se (VI) and As (III) and up to 154 ppm for other wastewater treatments.^63–65^

**Figure 2:**
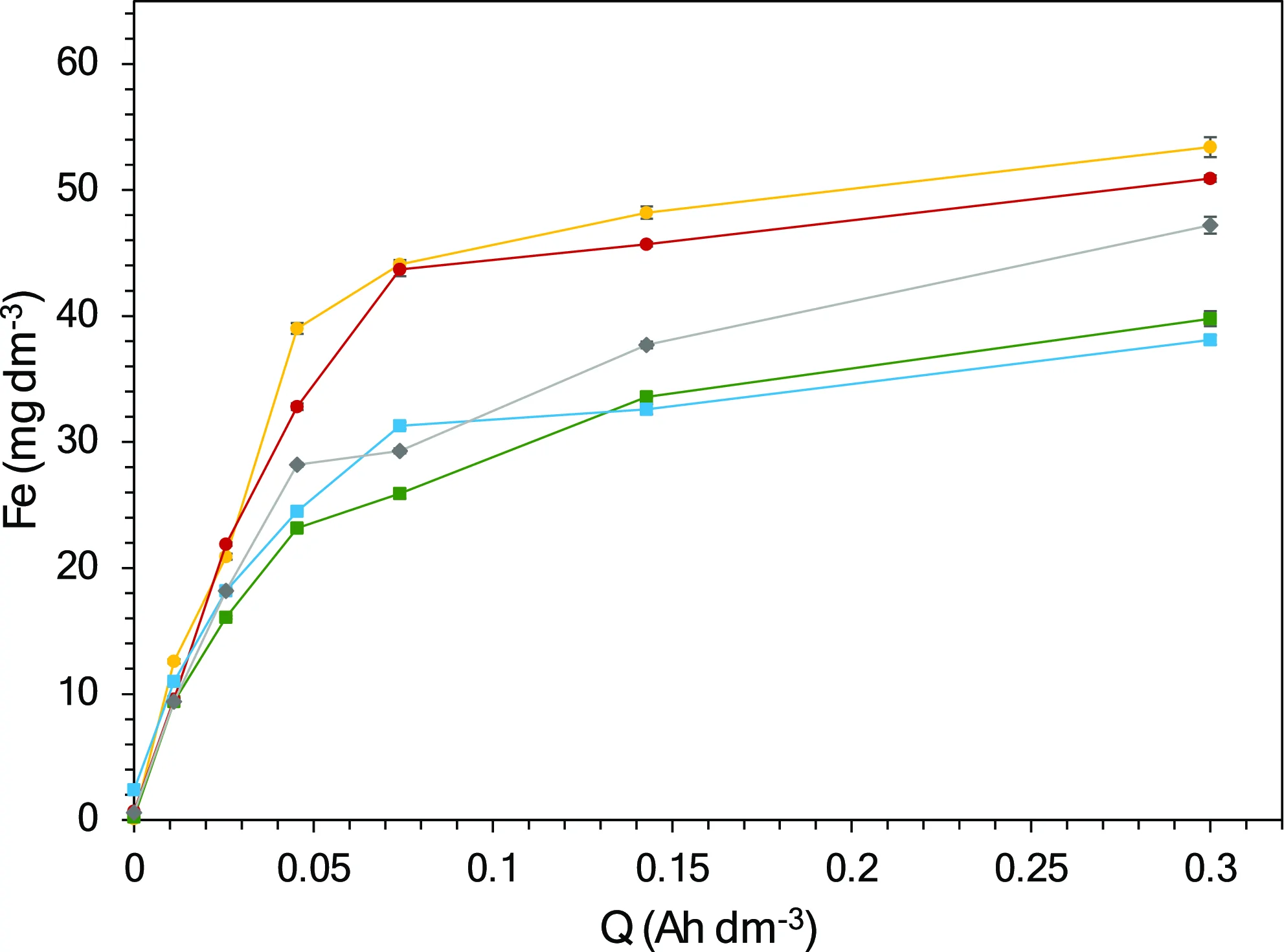
Evolution of Fe concentration dissolved from the anode with the applied electric charge. (● yellow) PS 43–53 μm; (■ green) PE 53–63 μm; (● red) PS 85–105 μm; (■ blue) PE 106–125 μm; and (◆ grey) blank. Operating conditions: j: 16.67 A m^–2^; *C*_0 electrolyte_: 0.01 M; *C*_0, MP_: 0.5 g L^–1^; interelectrode gap: 40 mm; *V*_tank_: 0.65 L; treatment time: 45 min; and anode: Fe. Error bars represent the standard deviation of triplicate measurements; note that most error bars are smaller than data points.

The coagulating strength of the Fe species generated in electrodissolution is reflected in the hydrolytic species distribution diagram, which relates pH to the predominant Fe species.^59,66^ Initial pH values were 7.6 ± 0.2 (PS 43–53 μm), 6.4 ± 0.1 (PE 53–63 μm), 7.4 ± 0.3 (PS 85–105 μm), and 9.4 ± 0.1 (PE 106–125 μm) with final values of 8.2 ± 0.4 (PS 43–53 μm), 8.8 ± 0.5 (PE 53–63 μm), 8.1 ± 0.4 (PS 85–105 μm), and 9.7 ± 0.1 (PE 106–125 μm), respectively (SI Figure 1S). The pH at maximum removal performance was 7.3 ± 0.3 (PS 43–53 μm), 8.2 ± 0.4 (PE 53–63 μm), 7.8 ± 0.5 (PS 85–105 μm), and 8.9 ± 0.1 (PE 106–125 μm) for the same groups. For the EC blank experiment, pH decreased from 9.0 ± 0.5 to 8.2 ± 0.4. The pH increase with applied electric charge in PS 43–53 μm, PE 53–63 μm, and PS 85–105 μm suggests the presence of polynuclear complexes and Fe­(OH)_3_, species known for high coagulation efficiency, likely contributing to effective removal at low charges. As pH evolves, Fe­(OH)_3_ formation becomes dominant, corresponding with maximum removal yields. However, the final pH favors a chemical environment where noncoagulating iron species coexist with Fe­(OH)_3_, contributing to the decline in MP removal (Figure 1). This pH increase results from hydrogen bubble generation at the cathode via water reduction, which dominates over acidic iron hydrolysis in initially neutral media. For PE 106–125 μm and EC blank systems, which start under alkaline conditions, rapid iron hydroxide formation may consume available OH^–^, causing pH to drop toward equilibrium. The subsequent pH increase in PE 106–125 μm systems likely reflects continuous OH^–^ generation exceeding consumption. These findings align with the literature, showing that pH trends converge toward neutrality, increasing when initial pH < 7.5 and decreasing under alkaline conditions (pH > 8.5).^67,68^ Note that to avoid chemical interference with the surfactant and allow EC to self-regulate pH dynamics, no external buffer was added. The low buffering capacity makes final pH readings more susceptible to intensified water electrolysis and chemical shifts from accumulated flocs.

Iron generation also affects the conductivity. The conductivity evolution with the applied electric charge (SI Figure 2S) shows minor oscillations caused by anode electrodissolution and formation of coagulating and noncoagulating species,^68–70^ while the high electrolyte concentration acts as a buffer, minimizing these fluctuations.

### 3.3 Zeta Potential and Coagulation Mechanisms

To investigate the dominant coagulation mechanisms, zeta potential evolution with the applied electric charge was measured for all MP systems (Figure 3). Initial zeta potentials were negative for all MPs: −10.5 ± 4.0 mV (PS 43–53 μm), −11.1 ± 1.5 mV (PE 53–63 μm), −13.1 ± 2.0 mV (PS 85–105 μm), and −14.6 ± 0.9 mV (PE 106–125 μm). Smaller MP particles exhibited less negative initial zeta potentials, possibly due to surfactant effects on the electrical double layer thickness.^71^ Their higher specific surface area may lead to denser adsorption of Tween 20.^72,73^ This nonionic layer, potentially synergizing with NaCl-induced electrical double layer compression,^74,75^ reduces the effective charge density detectable at the particle interface, explaining the size-dependent initial zeta potential differences observed.

**Figure 3:**
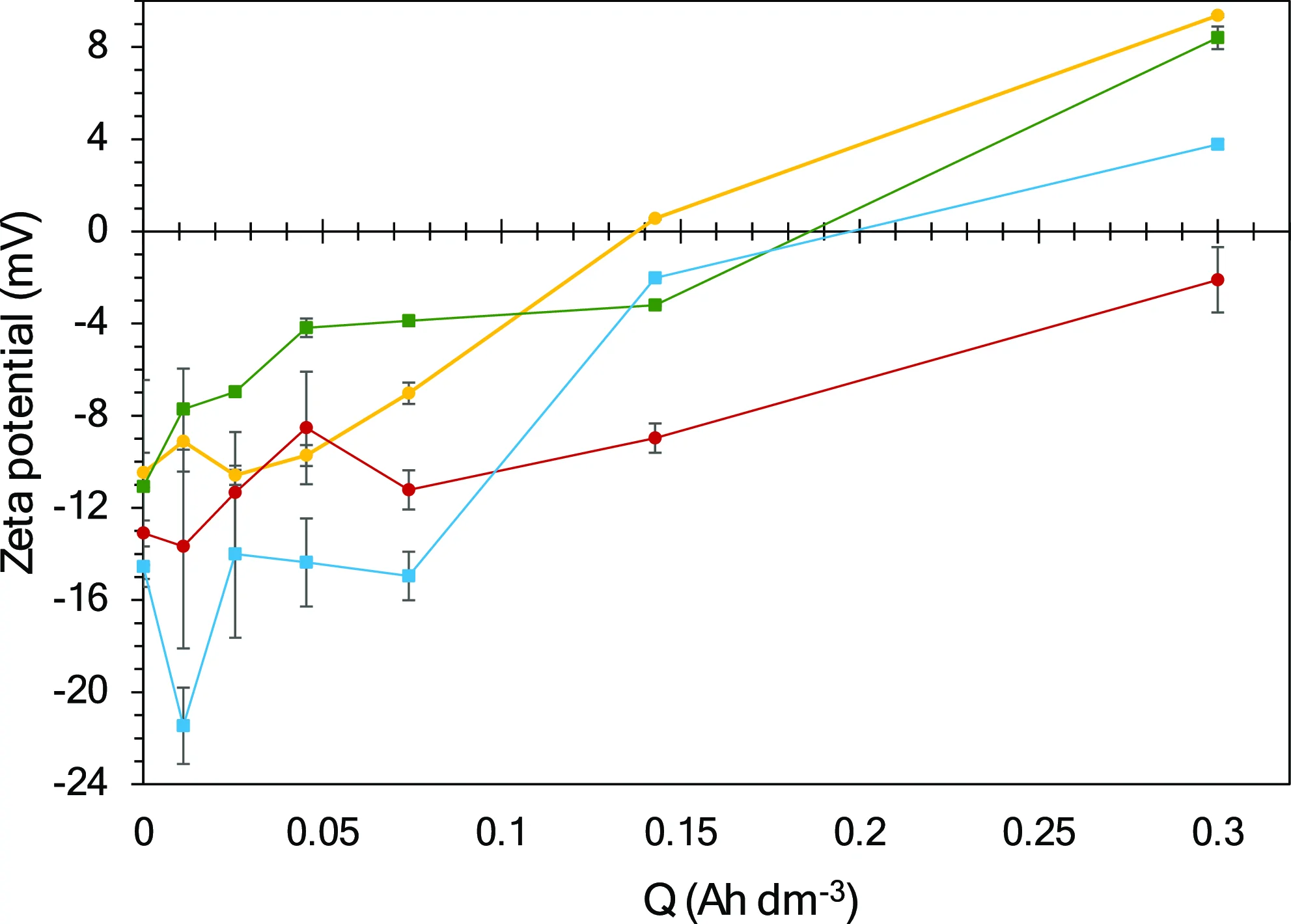
Evolution of the zeta potential with the applied electric charge. (● yellow) PS 43–53 μm; (■ green) PE 53–63 μm; (● red) PS 85–105 μm; and (■ blue) PE 106–125 μm. Operating conditions: j: 16.67 A m^–2^; *C*_0 electrolyte_: 0.01 M; *C*_0, MP_: 0.5 g L^–1^; interelectrode gap: 40 mm; *V*_tank_: 0.65 L; treatment time: 45 min; and anode: Fe.

With increasing applied electric charge, the zeta potential shifted toward positive values for most systems, except PS 85–105 μm, which remained slightly negative but close to zero. The zeta potential in colloidal systems indicates stability and interaction potential: more negative values (typically below −30 mV) suggest stable dispersions resistant to aggregation, while values near zero promote particle collision and aggregation. No clear trend emerged between PS and PE systems, but smaller particles showed higher zeta potentials at maximum MP removal and more positive final values compared to larger particles, likely due to their higher surface area-to-volume ratio facilitating coagulant interaction,^76^ enhanced by the combined effect of Tween 20 coating and NaCl-mediated double layer compression.

The pH values and zeta potentials at maximum removal efficiency reveal the dominant coagulation mechanisms.^66,77^ For PS systems, maximum removal occurred at pH 7.3 ± 0.3 (43–53 μm) and 7.8 ± 0.5 (85–105 μm), with the corresponding zeta potentials of −9.7 ± 0.5 mV and −8.5 ± 2.5 mV, respectively. These negative values, combined with the predominance of Fe­(OH)_3_ at these pH levels, indicate sweep flocculation^23,77^ as the primary removal mechanism for PS, likely complemented by charge neutralization as evidenced by the zeta potential progression. PE systems achieved maximum removal at pH 8.2 ± 0.5 (53–63 μm) and 8.9 ± 0.1 (106–125 μm), with near-zero zeta potentials of −3.2 ± 0.1 mV and −2.02 ± 0.03 mV, suggesting charge neutralization^23,77^ as the dominant mechanism by reducing interparticle repulsion. However, PE removal patterns at low applied electric charge indicate that sweep flocculation also contributes. Previous studies have identified sweep flocculation as the primary mechanism for both PS and PE removal in conventional coagulation with Al­(OH)_3_.^78^ Al is more prone to sweep flocculation due to its voluminous, gel-like precipitate, whereas Fe offers greater flexibility, enabling both sweep flocculation and charge neutralization depending on medium conditions.

### 3.4 Effects of the EC on MP Properties

Figure 4 shows a slight decrease in MP particle size from pristine particles to those noncoagulated (measured in the supernatant after sedimentation), regardless of the MP type or initial size. This reduction may be attributed to an electro-oxidative attack on the polymer surface.^53^ Tween 20 likely acts synergistically as a wetting agent, destabilizing the surface layer and increasing its susceptibility to strong oxidants such as hydroxyl radicals generated during EC. Size reduction was more pronounced for PS than for PE, likely due to PS’s higher interaction with the surfactant owing to its molecule structure. To identify possible chemical changes in MPs, FTIR spectra were recorded for individual components, Fe, PS, and PE, and for EC sludge mixtures (Fe-PS and Fe-PE) after treatment and sedimentation (SI Figure 3S). The characteristic peaks for each individual component are as follows:

- **Fe compounds**:^79–81^ 500 cm^–1^ (Fe–O bond), ∼1000 cm^–1^ (Fe–O–H stretching/deformation), and ∼3250 cm^–1^ (O–H stretching of hydroxyl groups). The 1620 cm^–1^ peak is absent due to sample drying.

- **PS**:^82^ 600 cm^–1^ (out-of-plane C–H deformation in the aromatic ring), 1490 and 1600 cm^–1^ (aromatic C = C stretching), 2850 cm^–1^ (aliphatic C–H stretching), and 3000 cm^–1^ (aromatic C–H stretching).
- **PE** ^83^ 750 cm^–1^ (CH_2_ rocking), 1500 cm^–1^ (CH_2_ bending), 2800 cm^–1^ (symmetrical CH_2_ stretching), and 2950 cm^–1^ (asymmetrical CH_2_ stretching).
  In the EC-treated samples, PS-EC spectra combine Fe and PS peaks, with an additional peak at 1200 cm^–1^, possibly from C = C bonds of alkenes and vinyl groups,^84^ suggesting slight PS ED-mediated oxidation. This is supported by a decrease in the carbonyl index.^53^ PE-EC spectra also show combined Fe and PE peaks, but Fe-related peaks shift slightly, indicating possible changes in Fe functional groups. Peak positions can vary: Fe–O and Fe–O–H bands range from 400 to 1100 ^81^ due to Fe species characteristics and O–H stretching appears around 3200–3400 depending on water retention.^85^ The 3500 cm^–1^ peak suggests that electrogenerated hydroxyl radicals attack the polymer backbone. A peak near 2300–2400 cm^–1^ observed in all EC likely originates from atmospheric CO_2_.^86^

**Figure 4:**
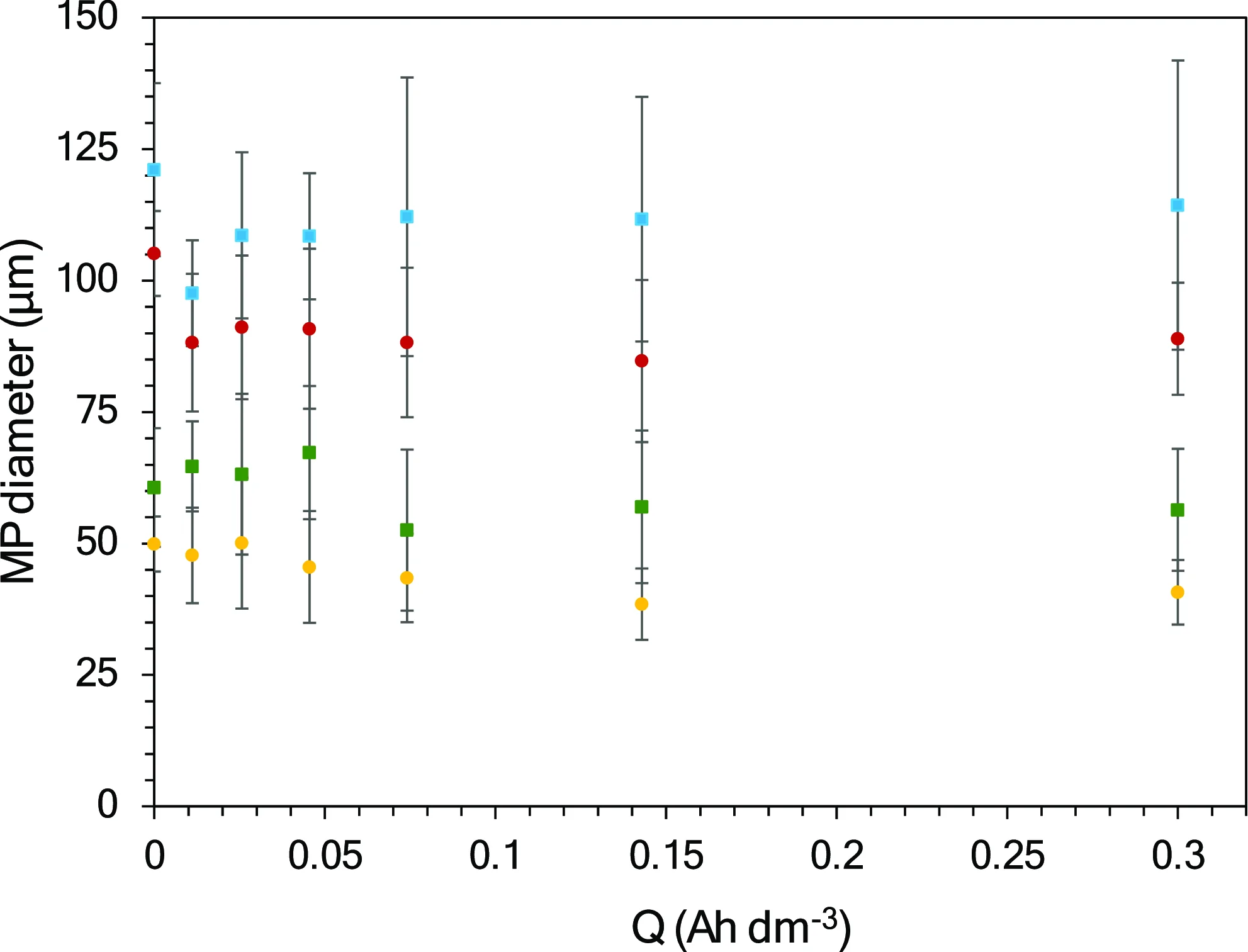
Changes in MP size with the applied electric charge. Noncoagulated MPs remaining in the supernatant of the samples from the bulk after sedimentation (● yellow) PS 43–53 μm; (■ green) PE 53–63 μm; (● red) PS 85–105 μm; and (■ blue) PE 106–125 μm. Operating conditions: j: 16.67 A m^–2^; C_0 electrolyte_: 0.01 M; *C*_0, MP_: 0.5 g L^–1^; interelectrode gap: 40 mm; *V*_tank_: 0.65 L; treatment time: 45 min; and anode: Fe. The data reflect the average diameter of all particles remaining in the supernatant measured with the MasterSizer 3000, and the error reflects the standard deviation.

### 3.5 Morphological Characterization of Floc-Microplastic Aggregates

Figure 5 presents key morphological parameters: diameter and growth factor. Average floc diameter increases with applied electric charge regardless of the MP type and size (Figure 5a–d), reaching a maximum at 0.045 Ah dm^–3^ (725 ± 316 μm (43–53 μm) and 842 ± 407 μm (85–105 μm) for PS and 779 ± 279 μm (53–63 μm) and 819 ± 450 μm (106–125 μm) for PE) before declining. MP particle size moderately affects floc diameter, with deviations caused by continuous floc evolution through aggregation and breakup. In both PS and PE systems, larger initial MP sizes produce larger floc diameters,^78^ as floc size directly correlates with particle size due to increased particle interactions^87^ and higher collision frequency and efficiency.^88^ In the blank experiment (Figure 5e), the maximum diameter (971 ± 257 μm) occurs later at 0.074 Ah dm^–3^ MPs, and iron hydroxides can polymerize into extensive unhindered networks,^89^ rather than fragmenting into smaller flocs through MP nucleation and termination effects. These measurements align with previous studies.^54^

**Figure 5:**
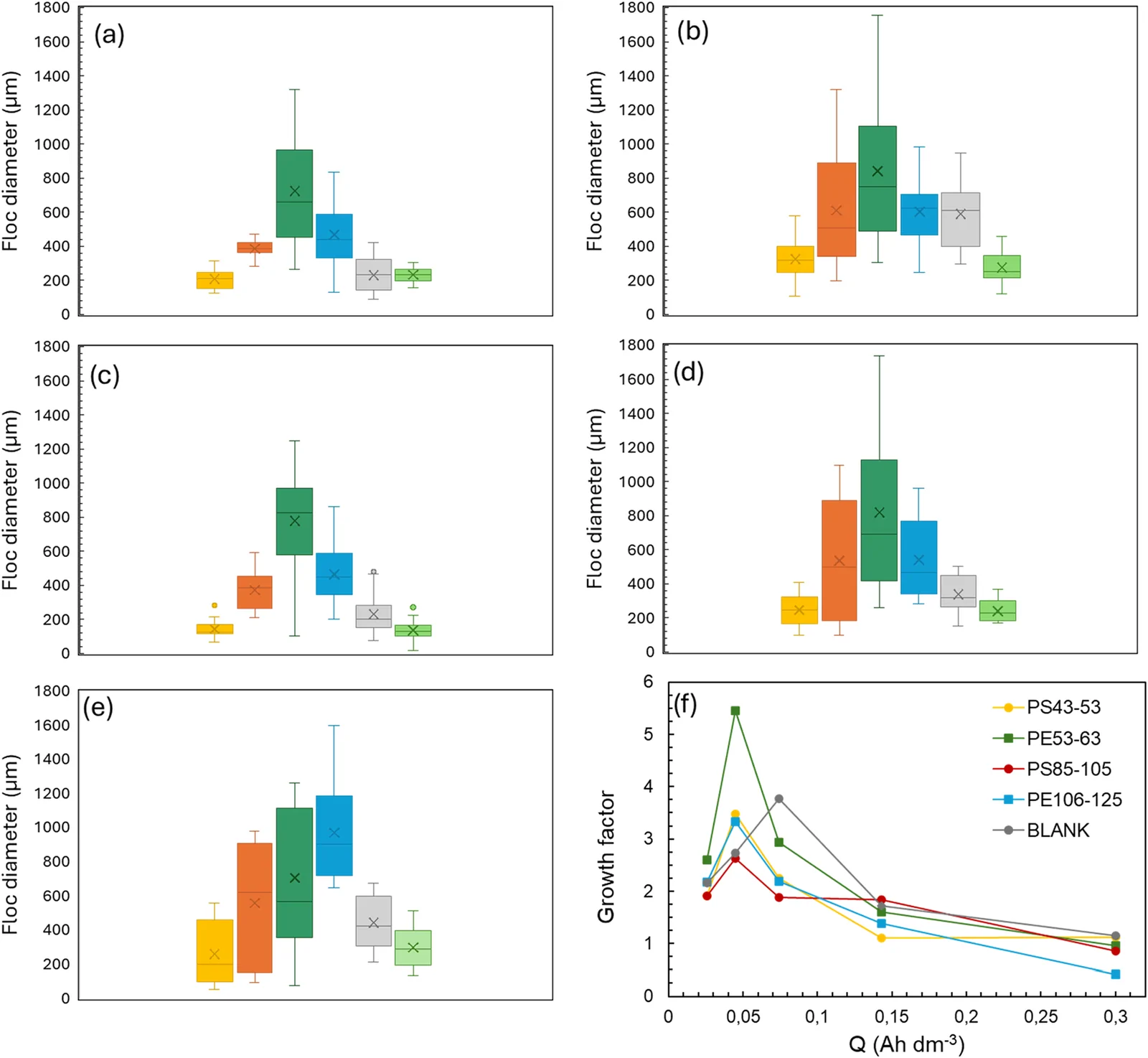
Changes in floc diameter of (a) PS 43–53 μm; (b) PS 85–105 μm; (c) PE 53–63 μm; and (d) PE 106–125 μm with applied electric charge. Legend: (yellow) 0.011 Ah dm^–3^; (orange) 0.026 Ah dm^–3^; (dark green) 0.045 Ah dm^–3^; (blue) 0.074 Ah dm^–3^; (light grey) 0.143 Ah dm^–3^; and (light green) 0.3 Ah dm^–3^. (e) Evolution of the growth factor, which was calculated with the average floc diameter, with applied electric charge.

For similar MP sizes, PS systems yield larger flocs than PE systems, attributed to differences in surface hydrophilicity and PS’s better affinity to coagulants.^54^

At maximum MP removal, PS systems formed larger flocs (726 ± 328 μm (43–53 μm) and 842 ± 415 μm (85–105 μm)) than PE systems (231 ± 103 μm (53–63 μm) and 340 ± 100 μm (106–125 μm)). This difference primarily reflects the applied electric charge at optimal removal: 0.045 Ah dm^–3^ for PS versus 0.143 Ah dm^–3^ for PE. The smaller floc sizes in PE systems align with their stable growth limit (Section 3.2). Larger flocs correlate with improved removal efficiency during sweep flocculation, consistent with previous studies.^78^ The literature shows that charge neutralization, dominant in PE systems, produces smaller, more fragile flocs,^90,91^ while sweep flocculation, prevalent in PS systems, generates larger, more robust flocs.^92^ Floc diameter also affects settling velocity (SI Figure 5S). For PS systems, maximum removal coincides with the highest sedimentation rate, while in PE systems, maximum removal does not correspond to the highest sedimentation velocity, suggesting that other factors influence MP removal efficiency.^93^ The EC-only system shows the lowest sedimentation rates, likely because pure coagulant flocs settle more slowly than similarly sized MP-coagulant aggregates. PS exhibits higher settling velocity than PE at the same applied charge, likely due to the larger floc size and its hydrophobic properties. PE is more nonpolar^53^ with lower surface energy than that of PS,^94^ making it more hydrophobic and thus more stable in suspension.^95^ Additionally, settleability is also related to floc density. Figure 2 shows that the concentration of dissolved iron at the same applied charge is higher for PS systems than for PE systems, and the density of PS is slightly higher than that of PE. Therefore, it can be suggested that PS-EC flocs have higher density than that of PE-EC flocs.

The growth factor, reflecting floc development and rupture, follows a similar trend (Figure 5f) with its limit appearing near 0.045 Ah dm^–3^ ^40,96^ and a higher limit in blank experiments (0.074 Ah dm^–3^). Smaller flocs resist breakage better than larger ones,^88^ due to unstable MP-floc aggregation,^43^ cohesive forces overcoming floc strength,^43,97^ surface erosion,^88^ and the weaker structure of large flocs.^97^

Fractal dimension relates to the complexity and density of flocs’ internal geometric structures.^98^ Figure 6a shows its evolution with applied electric charge for both MP types and blanks. Initially, the fractal dimension increases with the charge, indicating a transition from open, branched to compact flocs. PS systems increase from 1.61 to 1.65 to 1.86–1.87 at 0.074 Ah dm^–3^ and then remain constant.^99^ PE systems increase from 1.58 to 1.83 to 1.78–1.91 at 0.045 Ah dm^–3^, but at higher charges, the fractal dimension decreases to steady-state values (1.72–1.77), linked to exceeding floc strength due to increased compaction-related bonds.^99^ PS maintains constant fractal dimension despite decreasing diameter, suggesting restructuring with stable internal compaction.^87^ The literature confirms a direct relationship between the fractal dimension and floc diameter^100^ and that compact flocs with a high fractal dimension can form from combining irregular flocs with low fractal dimension.^88^ Floc breakage into more symmetrical structures reduces the fractal dimension,^99^ with surface erosion driving the fragmentation of compact flocs.^99^

**Figure 6:**
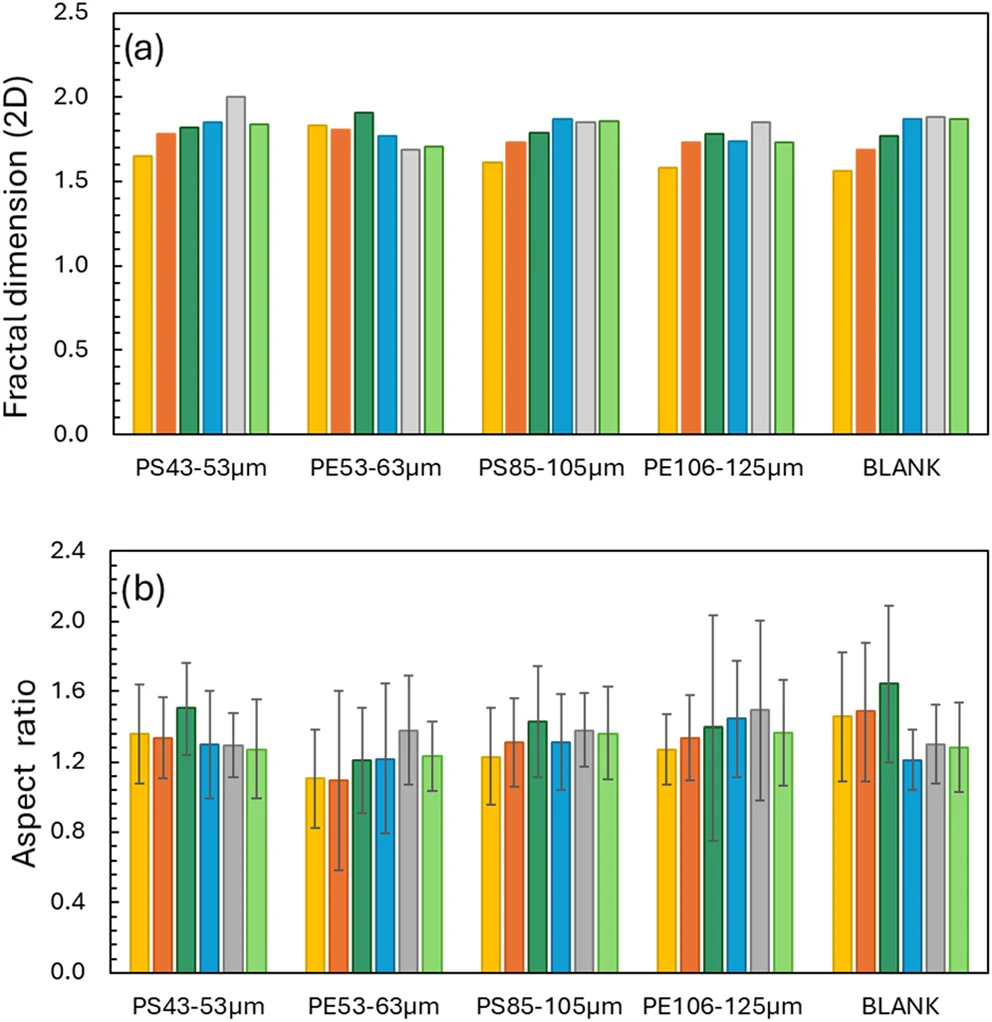
Changes in (a) two-dimensional fractal dimension (*r* ^2^ > 0.99) and (b) aspect ratio with applied electric charge. Legend: (yellow) 0.011 Ah dm^–3^; (orange) 0.026 Ah dm^–3^; (dark green) 0.045 Ah dm^–3^; (blue) 0.074 Ah dm^–3^; (light grey) 0.143 Ah dm^–3^; and (light green) 0.3 Ah dm^–3^.

For the same MP type, larger initial MP sizes yield lower final fractal dimensions. Larger flocs increase MP-floc hydrodynamic force differences, expanding the shear plane.^43^ PE systems exhibit lower final fractal dimensions than those of PS, reflecting different coagulation mechanisms.^91^ EC under charge neutralization produces flocs with a lower fractal dimension than that of sweep flocculation.^91^

The aspect ratio complements the fractal dimension by describing the floc shape. Values near 1 indicate a spherical shape, while higher values indicate irregular, elongated shapes. Figure 6b shows that the aspect ratio increases with the applied electric charge until 0.045 Ah dm^–3^ for PS and blanks and 0.143 Ah dm^–3^ for PE. This increase reflects elongation concurrent with growth in diameter and fractal dimension, consistent with cluster–cluster aggregation,^99^ until flocs reach a structural limit and become highly intricate.^98^ Subsequent rupture smooths irregular shapes^88,99^ but does not produce spherical flocs.

PS systems exhibit higher aspect ratios than those of PE, possibly due to stronger MP–Fe interactions in PS. Flocs larger than 200 μm typically have irregular shapes.^43^ Combined parameters suggest that PS forms compact but elongated flocs, while PE produces fluffier, more symmetrical flocs. Floc structure images (SI Figures 6S-10S) show the evolution of the flocs: initial aggregation followed by the breakup into smaller flocs with excess iron at high applied charges. These images confirm greater symmetry in PE-EC flocs and greater elongation in PS-EC flocs.

## 4 Conclusions

This study evaluates electrocoagulation (EC) for removing microplastics (MPs), focusing on the effects of the MP type and size on removal efficiency, as well as the interaction between MPs and flocs.

– EC effectively removes polystyrene (PS) and polyethylene (PE) MP. Maximum PS removal rates were achieved at 0.045 Ah dm^–3^: 73.0 ± 0.3% and 80.6 ± 4.5% for PS of 43–53 μm and 85–105 μm, respectively. Maximum PE removal was achieved at 0.143 Ah dm^–3^: 71.8 ± 4.7% for 53–63 μm PE and 79.1 ± 3.3% for 106–125 μm PE. PS removal was consistently higher than that of PE, likely due to PS’s greater surface hydrophilicity, interaction with coagulation species, and associated floc formation mechanisms. Larger pristine MPs exhibited better removal, correlating with larger floc sizes and faster sedimentation rates.
– MP removal efficiency increases with the applied electric charge up to a peak (0.045 Ah dm^–3^ for PS and 0.143 Ah dm^–3^ for PE), after which it declines regardless of the MP type or size. This decrease is attributed to the initial formation of effective coagulant species at low charges transitioning to less effective species at higher charges as pH changes, alongside anode passivation and floc breakage.
– The dominant EC mechanism differs by the MP type: sweep flocculation primarily governs PS removal, while charge neutralization dominates PE removal, as indicated by pH and ζ-potential measurements. This mechanistic difference influences floc characteristics with sweep flocculation producing larger, more robust flocs. Additionally, sweep flocculation requires less energy and iron dosage than those for charge neutralization.
– Zeta potential is influenced by the specific surface of MPs and by the presence of a surfactant and a salt electrolyte.
– The evolution of the uncoagulated MP size suggests the presence of electroactive species that slightly modify the surface, as reflected in FTIR analysis with a new peak at 1200 cm^–1^ for PS and another one at 3500 cm^–1^ for PE.
– The Floc diameter, fractal dimension, and aspect ratio evolutions relate to both MP–coagulant affinity and the main EC mechanism, determining the floc morphology. PS-EC forms more compact and elongated flocs, whereas PE-EC produces fluffier, more symmetrical flocs. Additionally, the floc size positively correlates with the pristine MP size.
– A limit in floc growth occurs at an applied electrical charge of 0.045 Ah dm^–3^, while internal geometry (fractal density) continues to evolve at higher charges, before reaching a steady state. The maximum floc size does not guarantee the maximum MP removal, as this depends on other factors such as MP-Fe interactions and the EC mechanism.

On the basis of the results of this work, future research will assess how floc kinetics and interaction mechanisms change with both simulated complex and real-world matrices containing weathered MPs of different origins and shapes. In addition, the transition to continuous EC will enable this study to be carried out under realistic hydrodynamic conditions.

## Supplementary Material



## Notes

Evolution of the pH and the conductivity with the applied electric charge for all experiments; FTIR spectra of Fe, PS, PE, and final EC sludge samples for all the experiments; evolution of settling velocity with the average floc diameter; evolution of the images obtained of initial wastewater to be treated, flocs during settling, and supernatant/clarified water after EC from the image-capturing device with the applied electric charge for all the experiments (PDF)
