# Supplementary material for: Electrocoagulation for Polystyrene and Polyethylene Microplastic Removal: Particle Size Effects, Mechanisms, and Floc Characterization
Source: ACS ES T Water. 2026 Jul 28;6(8):5132–42. doi: 10.1021/acsestwater.6c00398 (PMC13488408; doi:10.1021/acsestwater.6c00398)
Supplement: Supplementary file 1 [file ew6c00398_si_001.pdf]

Electrocoagulation for Polystyrene and Polyethylene Microplastic Removal: Particle Size Effects, Mechanisms, and Floc Characterization

Sara Mateo [a,*], Jannis Wenk [b,c], John Chew [b], Antonio Exposito [b].

[a] Department of Chemical and Materials Engineering, Complutense University of Madrid, Madrid, 28040, Spain

[b] Department of Chemical Engineering, University of Bath, Bath, BA2 7AY, United Kingdom

[c] Division G - Qualitative Hydrology, Department G1 - General Water Quality Issues. Federal Institute of Hydrology, Koblenz, 56068, Germany

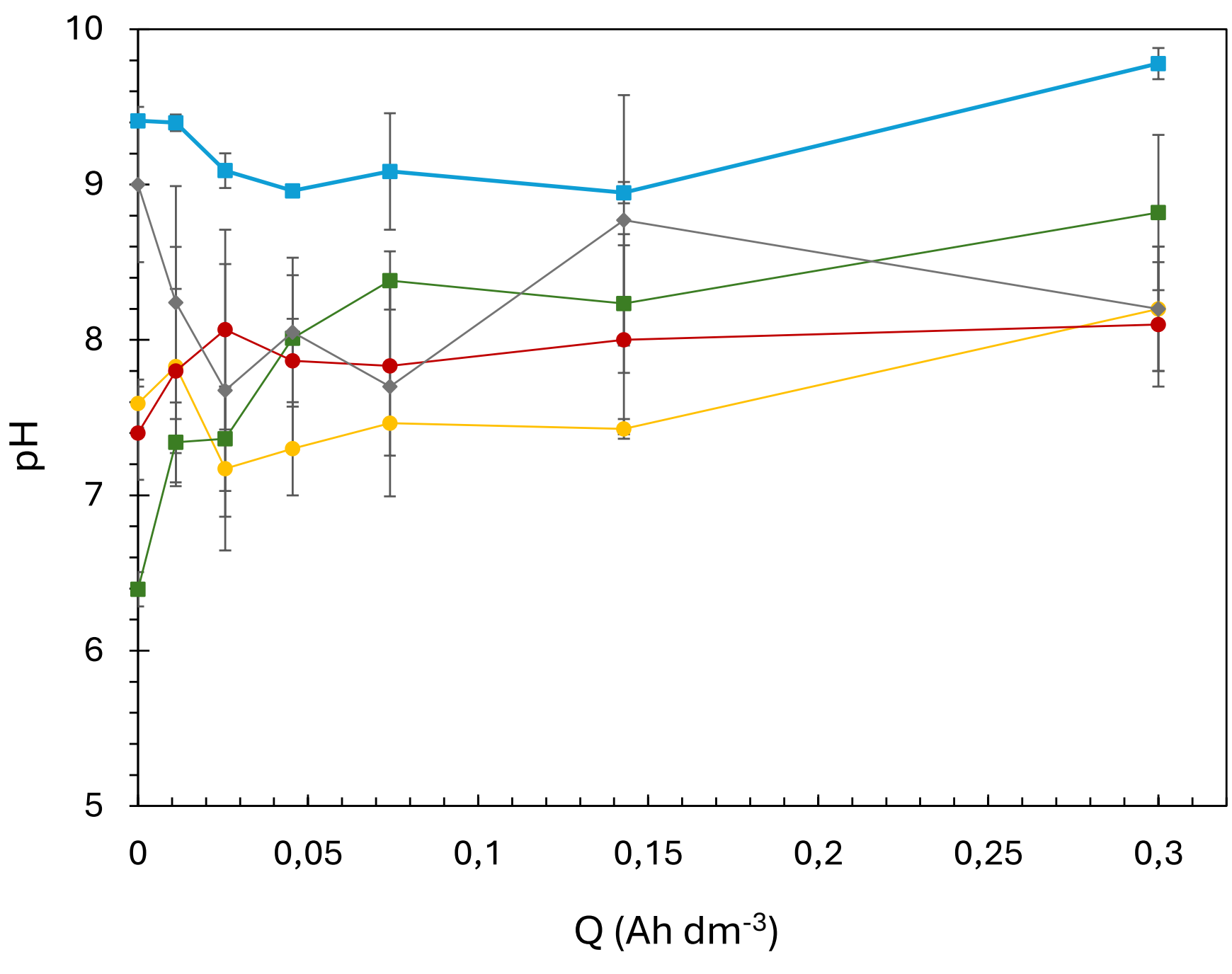

Figure 1S. Evolution of the pH with the applied electric charge. (●) PS 43-53 µm; (■) PE 53-63 µm; (●) PS 85-105 µm; (■) PE 106-125 µm; (◆) Blank. Operating conditions: j: 16.67 A m^{-2}; $C_{0\ electrolyte}$: 0.01 M; $C_{0,\ MP}$: 0.5 g L^{-1}; Inter-electrode gap: 40 mm; V_{tank}: 0.65 L; Treatment time: 45 min; Anode: Fe. Error bars represent the standard deviation of triplicate measurements

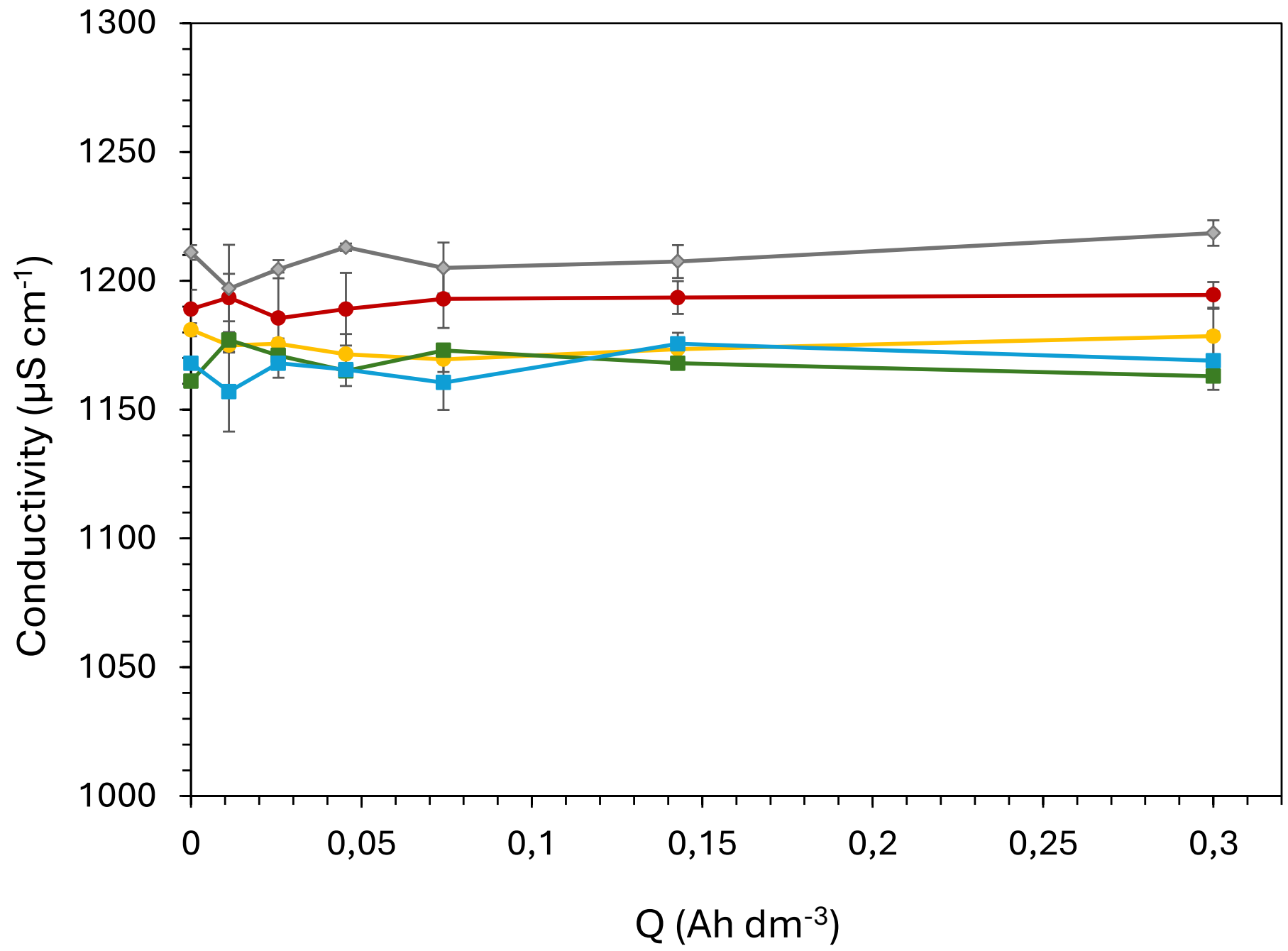

Figure 2S. Evolution of the conductivity with the applied electric charge (●) PS 43-53 µm; (■) PE 53-63 µm; (●) PS 85-105 µm; (■) PE 106-125 µm; (◆) Blank. Operating conditions: j: 16.67 A m^{-2}; $C_{0\ electrolyte}$: 0.01 M; $C_{0,\ MP}$: 0.5 g L^{-1}; Inter-electrode gap: 40 mm; V_{tank}: 0.65 L; Treatment time: 45 min; Anode: Fe. Error bars represent the standard deviation of triplicate measurements

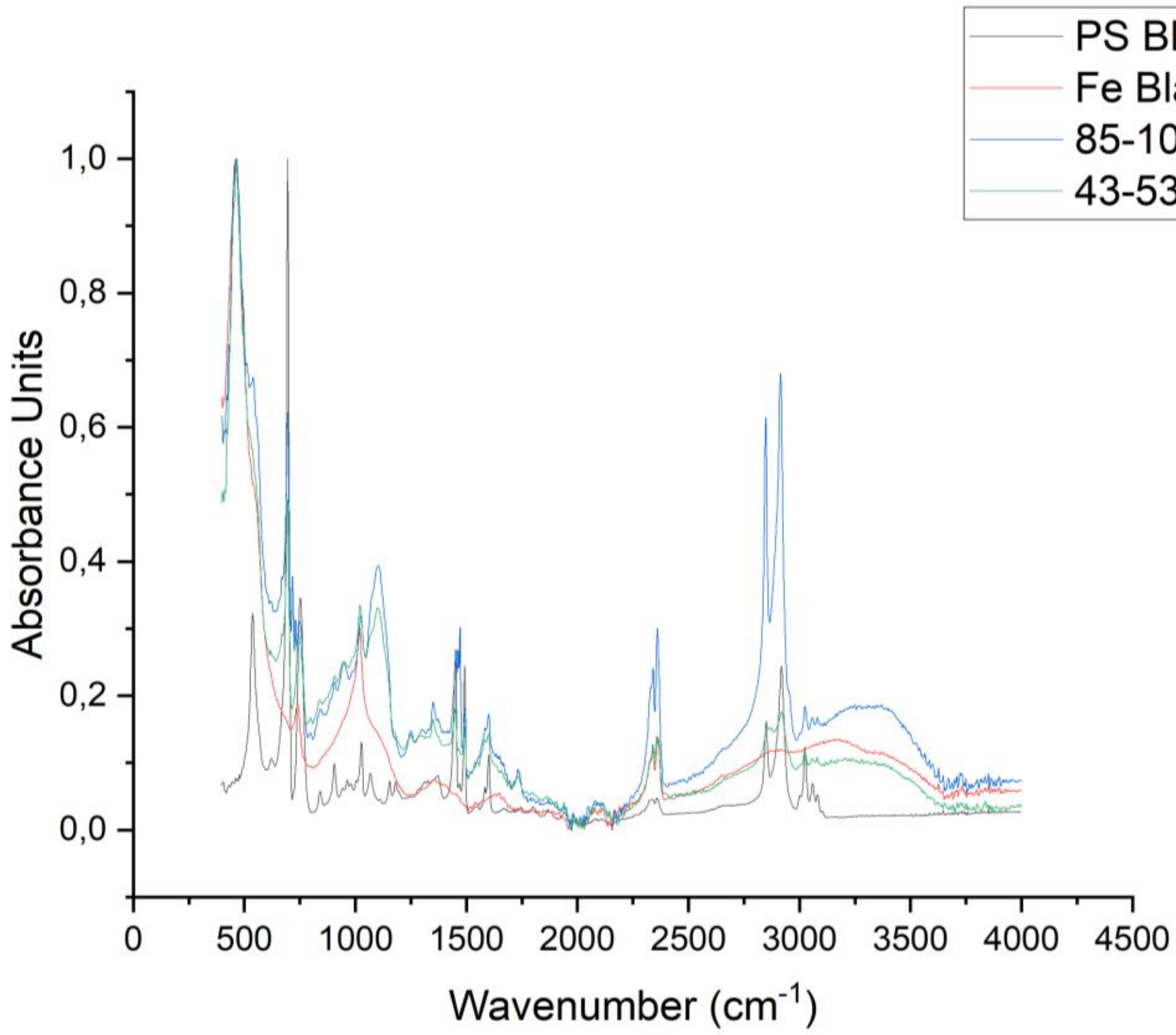

Figure 3S. FTIR spectra for PS-EC.

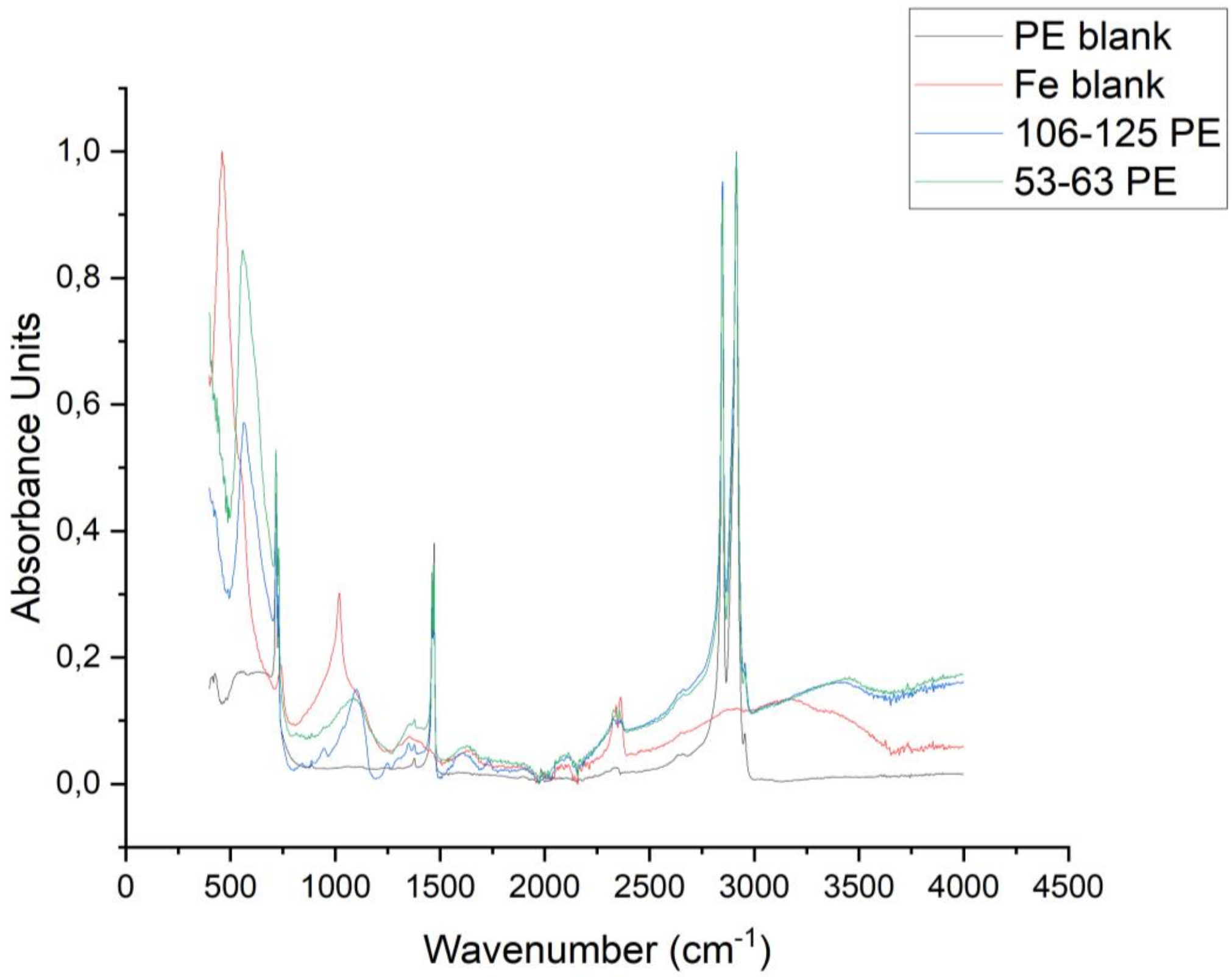

Figure 4S. FTIR spectra for PE-EC.

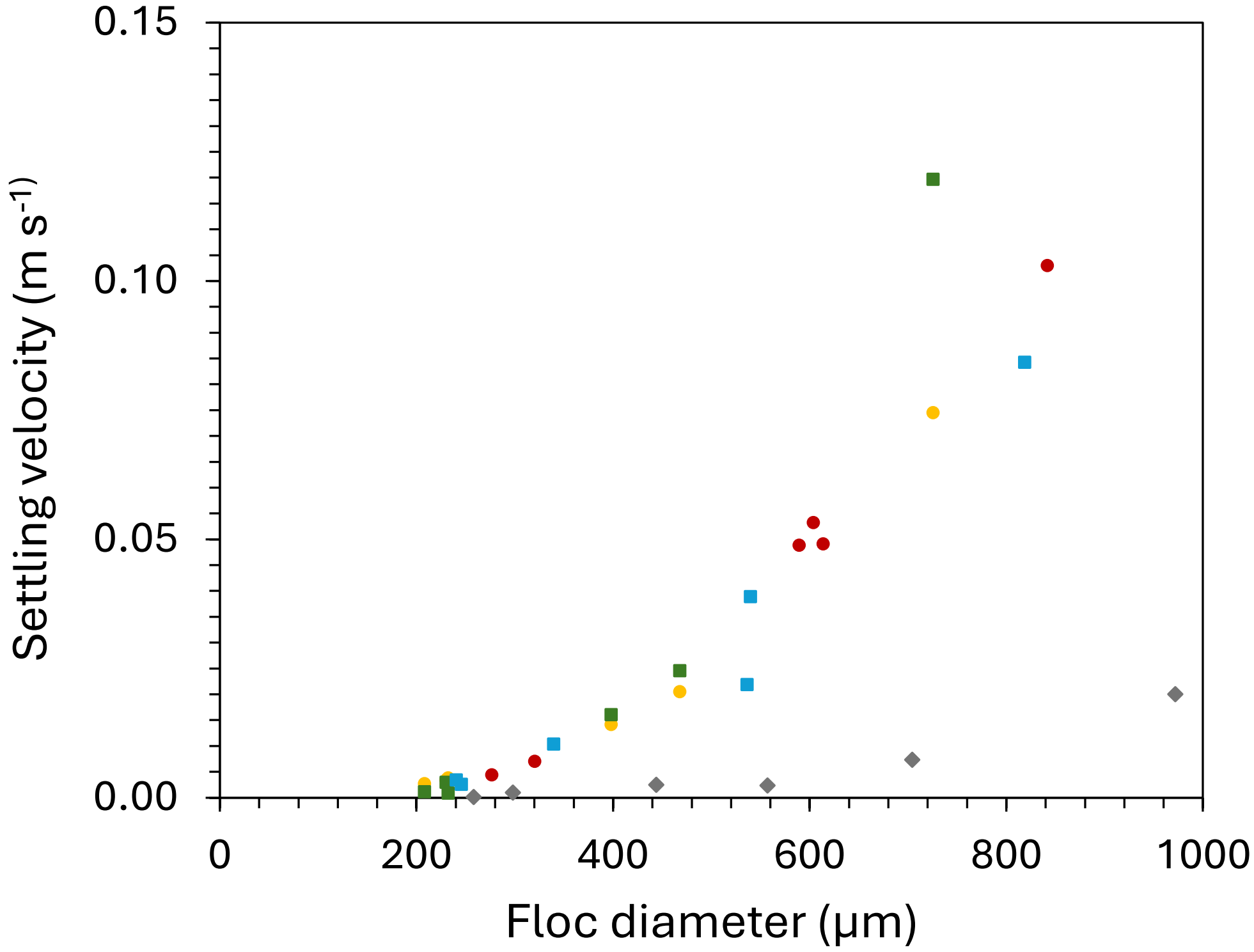

Figure 5S. Evolution of settling velocity with average floc diameter. (●) PS 43-53 µm; (■) PE 53-63 µm; (●) PS 85-105 µm; (■) PE 106-125 µm. (◆) Blank. Operating conditions: j: 16.67 A m^{-2}; $C_{0\ electrolyte}$: 0.01 M; $C_{0,\ MP}$: 0.5 g L^{-1}; Inter-electrode gap: 40 mm; V_{tank}: 0.65 L; Treatment time: 45 min; Anode: Fe.

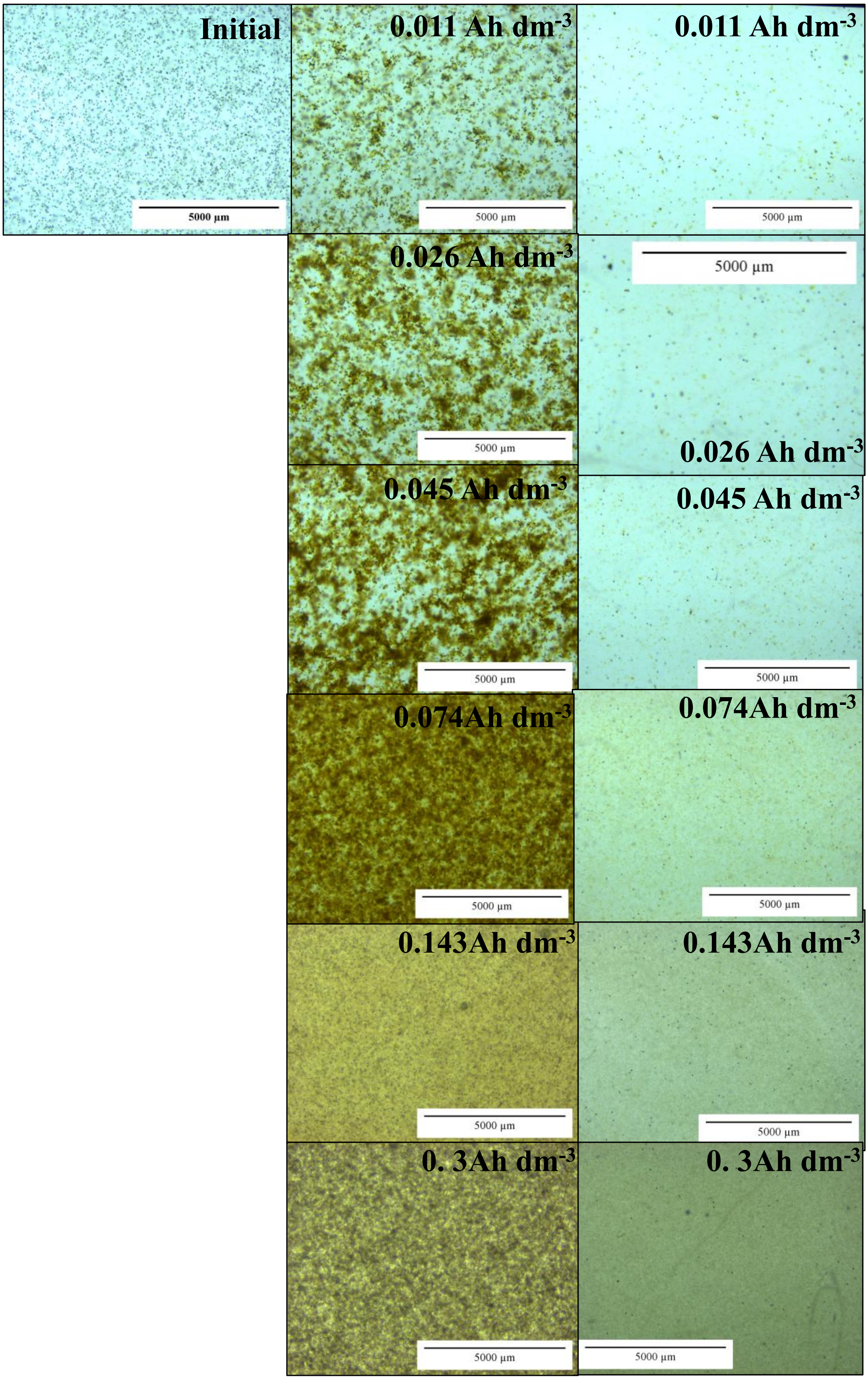

Figure 6S. Evolution of the images obtained from the image capturing device with the applied electric charge for 43-53 µm PS-EC. Initial wastewater to be treated, flocs during settling and supernatant/clarified water after EC.

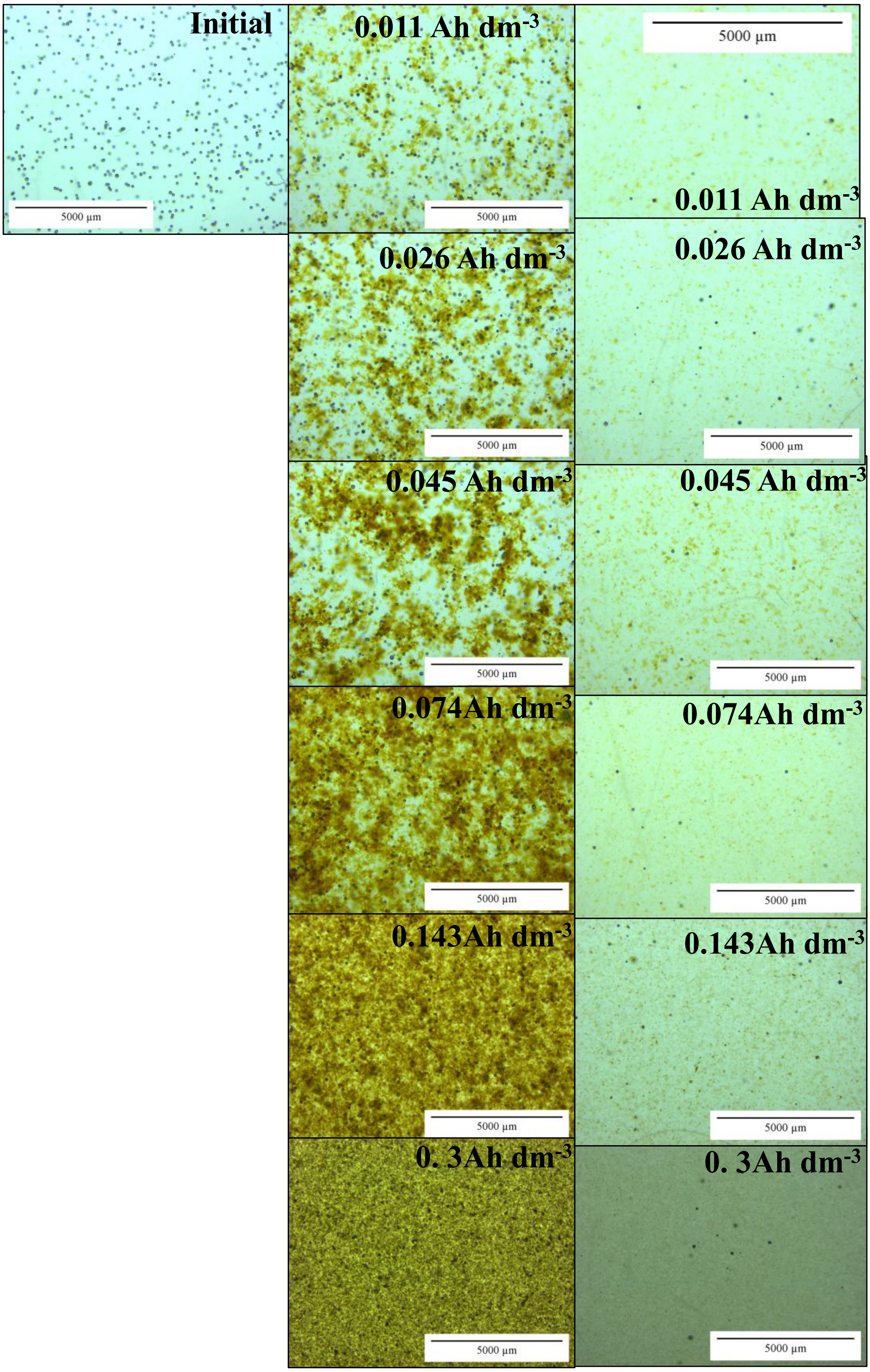

Figure 7S. Evolution of the images obtained from the image capturing device with the applied electric charge for 85-105 µm PS-EC. Initial wastewater to be treated, flocs during settling and supernatant/clarified water after EC.

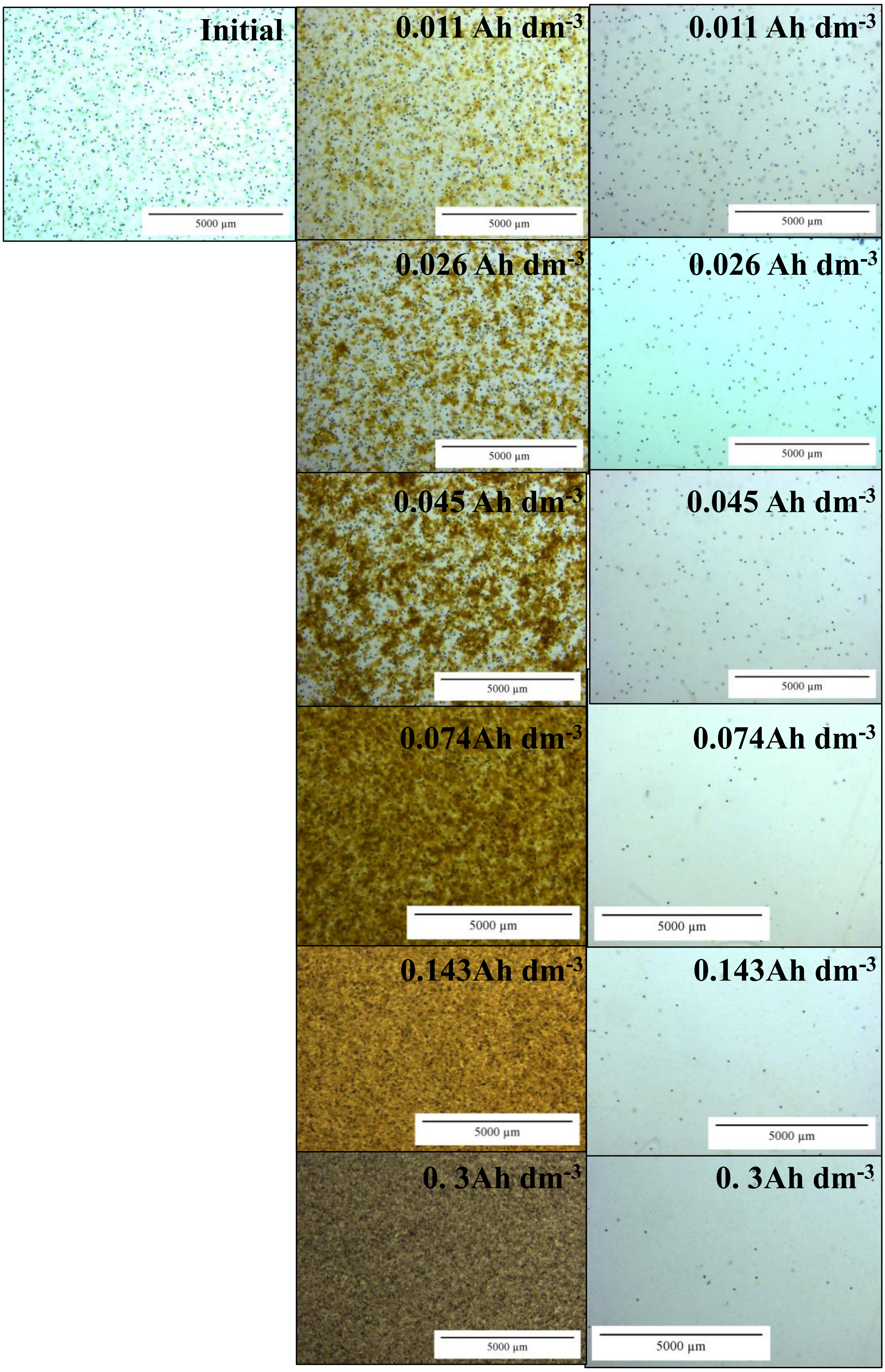

Figure 8S. Evolution of the images obtained from the image capturing device with the applied electric charge for 53-63 µm PE-EC. Initial wastewater to be treated, flocs during settling and supernatant/clarified water after EC.

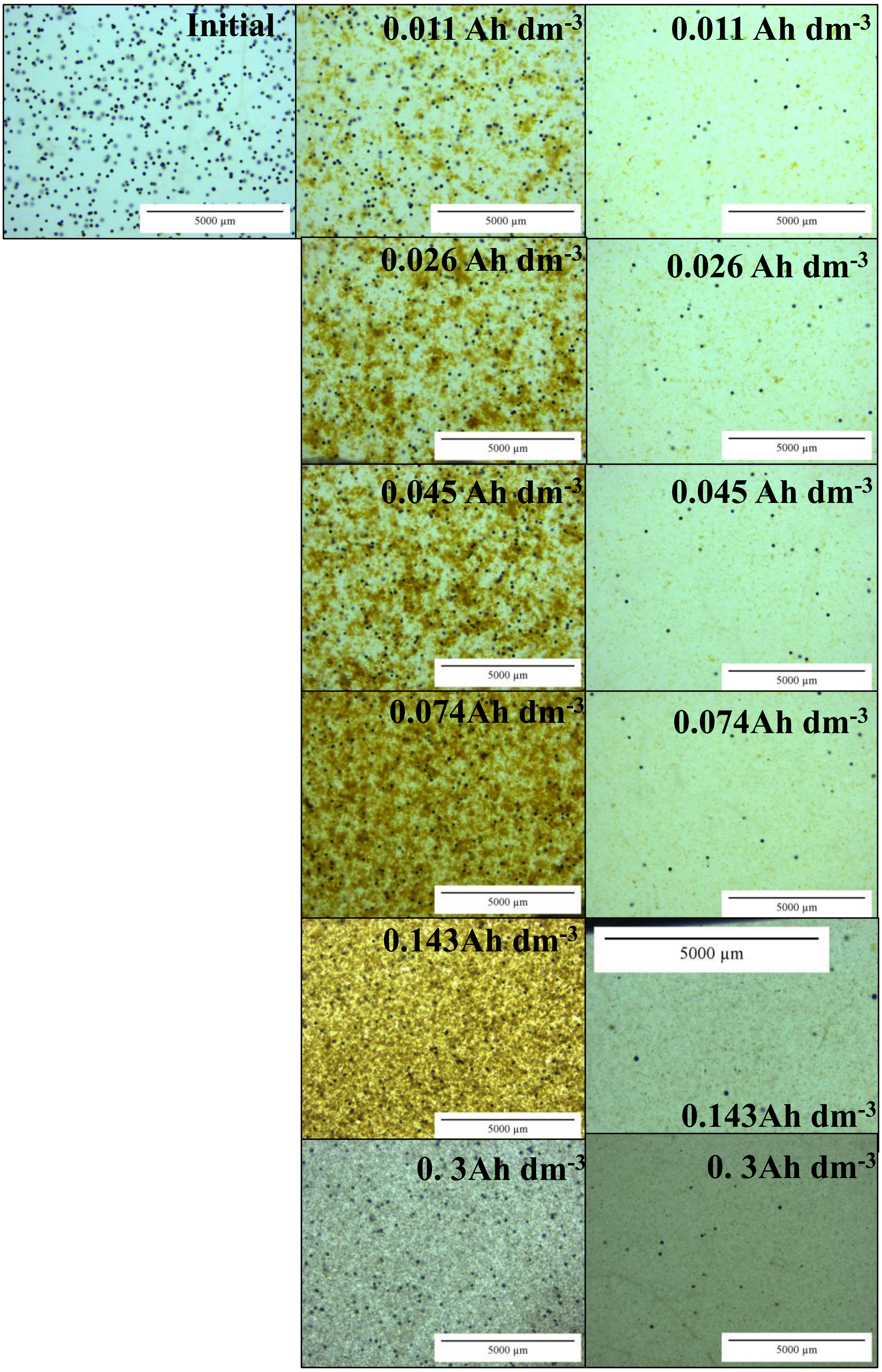

Figure 9S. Evolution of the images obtained from the image capturing device with the applied electric charge for 106-125 µm PE-EC. Initial wastewater to be treated, flocs during settling and supernatant/clarified water after EC.

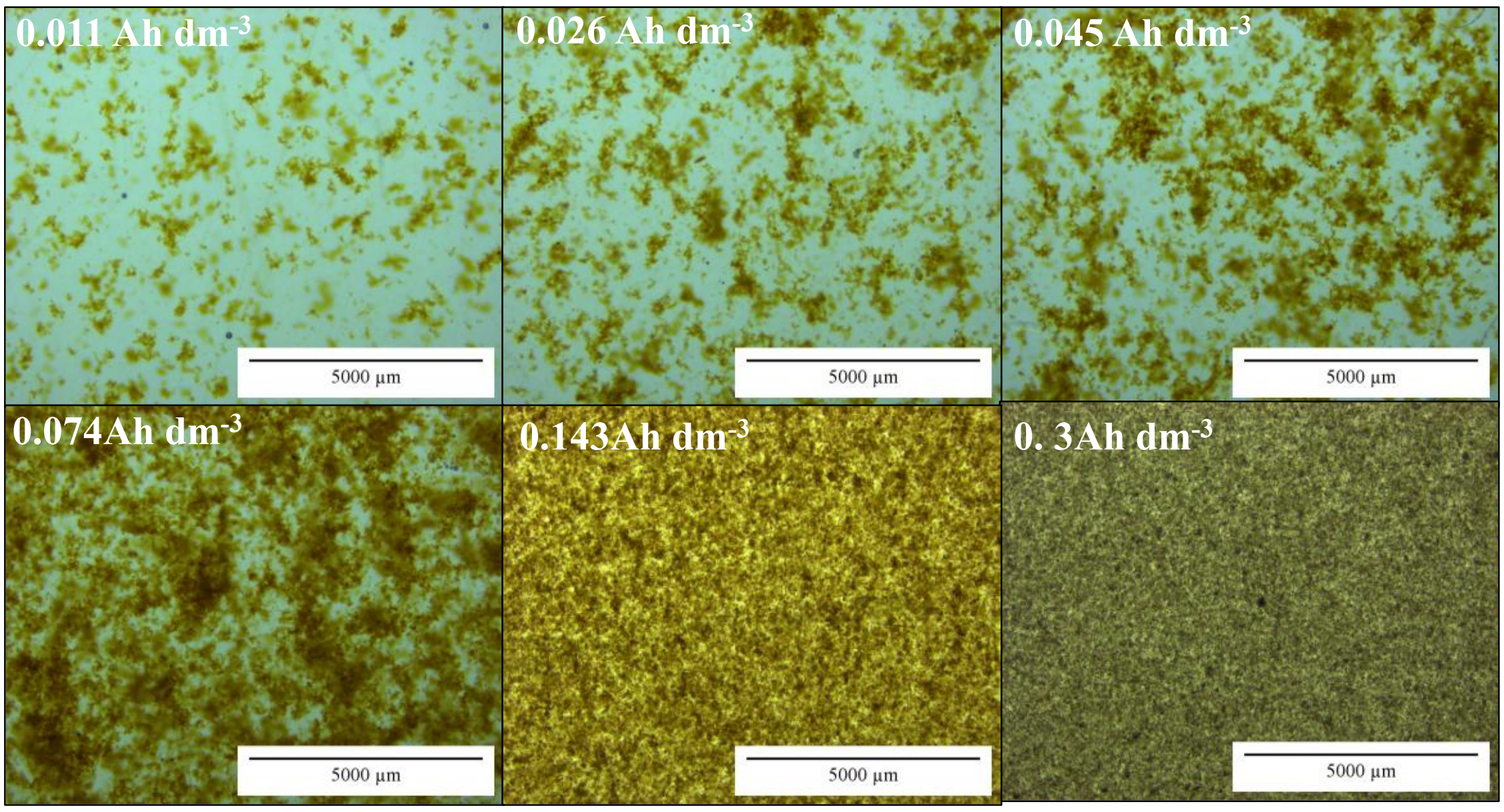

Figure 10S. Evolution of the images obtained from the image capturing device with the applied electric charge for blank-EC during the flocs sedimentation.
